# Mutation of two key aspartate residues alters stoichiometry of the NhaB Na^+^/H^+^ exchanger from *Klebsiella pneumoniae*

**DOI:** 10.1038/s41598-019-51887-2

**Published:** 2019-10-28

**Authors:** Miyer Patiño-Ruiz, Klaus Fendler, Octavian Călinescu

**Affiliations:** 1 Department of Biophysical Chemistry, Max Planck Institute of Biophysics, Frankfurt am Main, Germany; 2 Department of Biophysics, Carol Davila University of Medicine and Pharmacy, Bucharest, Romania

**Keywords:** Permeation and transport, Membrane proteins

## Abstract

Bacterial NhaB Na^+^/H^+^ exchangers belonging to the Ion Transporter superfamily are poorly characterized in contrast to Na^+^/H^+^ exchangers of the Cation Proton Antiporter superfamily which have NhaA from *Escherichia coli* as a prominent member. For a more detailed understanding of the intricacies of the exchanger’s transport mechanism, mutational studies are essential. Therefore, we mutated two protonatable residues present in the putative transmembrane region of NhaB from *Klebsiella pneumoniae* (KpNhaB), which could serve as substrate binding sites, Asp146 and Asp404, to either glutamate or alanine and analyzed transport function and stability of the mutants using electrophysiological and fluorimetric techniques. While mutation of either Asp residue to Glu only had slight to moderate effects on the transport activity of the exchanger, the mutations D404A and D146A, in particular, had more profound effects on the transport function. Furthermore, a double mutant, D146A/D404A, exhibited a remarkable behavior at alkaline pH, where recorded electrical currents changed polarity, showing steady-state transport with a stoichiometry of H^+^:Na^+^ < 1, as opposed to the H^+^:Na^+^ > 1 stoichiometry of the WT. Thus, we showed that Asp146 and Asp404 are part of the substrate binding site(s) of KpNhaB and engineered a Na^+^/H^+^ exchanger with a variable stoichiometry.

## Introduction

The existence of ion concentration gradients is a characteristic of living cells, which rely on these gradients to drive essential cellular processes, to establish their membrane potential and to control their cell volume. Consequently, all organisms require strict control of their intracellular ion concentrations, which is established by specialized transport systems in their plasma membrane. Two of the major ions that require strict homeostatic control are H^+^ and Na^+^ ^1,2^. pH values outside the normal physiological range have a deleterious effect on enzymatic reactions, while too low or excessive amounts of Na^+^ are stressors to the cell^3^.

A major system involved in Na^+^ and pH homeostasis is represented by the Na^+^/H^+^ exchangers or antiporters^3–5^, which serve two major roles – either they are specialized for using a pre-existing Na^+^ gradient to remove excessive H^+^ from the cell, as is the case of the human NHE exchangers^6^, or they are specialized in removing excessive intracellular Na^+^, as is the case of bacterial NhaA or NhaB exchangers^7,8^. By far the best characterized Na^+^/H^+^ exchanger is NhaA from *Escherichia coli* (EcNhaA), the first Na^+^/H^+^ exchanger which was crystallized^9^. According to the Transporter Classification Database^10^, EcNhaA belongs to the Cation Proton Antiporter Superfamily (CPA), a transport family that also includes the human NHE exchangers, as well as the NapA and NhaP proteins, for which representative members have recently had their structures resolved^11–13^.

Despite the wealth of information available on CPA members, much less is known about non-CPA Na^+^/H^+^ exchangers, such as NhaB proteins, which are members of the Ion Transporter (IT) superfamily^14^ and share almost no sequence similarity to NhaA-class proteins^15^. NhaB-encoding genes are present in the genomes of Gram-negative bacteria^14^ and it has been shown that, in *E. coli*, NhaB is indispensable under conditions where NhaA is either not expressed or has low activity (low pH or low Na^+^ concentration)^8^. In *Y. pestis*, NhaB, together with NhaA, have been shown as essential for virulence^16^.

No structural information is, as of this moment, available regarding NhaB antiporters, to the point where even the number of transmembrane helices (TMs) that they possess has not been established conclusively. Thus, despite hydropathy plots indicating a number of 12^15^ or 13 TMs^17^, a topological study on NhaB of *V. alginolyticus* (VaNhaB) proposed the presence of only 9 TMs^17^. Functionally, slightly more information is available. Unlike NhaA, which catalyzes H^+^:Na^+^ exchange at a 2:1 ratio^7^, NhaB has a 3:2 stoichiometry^18^. A small number of functional studies have been performed on NhaB family members, relying mainly on fluorescence dequenching methods^19–23^. Since NhaB is an electrogenic transporter, it is well adapted to characterization by electrophysiological techniques, in particular solid supported membrane (SSM)-based electrophysiology^24^. We have recently characterized^25^ a member of the NhaB family, NhaB from *Klebsiella pneumoniae* (KpNhaB) and were able to show that, despite the absence of sequence similarity to CPA exchangers, the function of KpNhaB can be described by a similar competition-based mechanism^25,26^.

As protonatable residues are essential for Na^+^/H^+^ exchanger function^3^ we sought to identify similarly charged residues in the putative TMs of KpNhaB, as determined by alignment^25^ with the TMs established by the previous topological study on VaNhaB^17^. Two such residues are, in the sequence of KpNhaB, Asp146 and Asp404. A previous study^21^ performed a mutational analysis on Asp147 from VaNhaB, the homologous residue of Asp146. Its conclusion was that Asp147 is essential for the function of the antiporter, as replacement of this residue with Gly, Glu, or Thr resulted in the abolishment of Na^+^/H^+^ exchange, though not in the loss of ^22^Na^+^/Na^+^ exchange in VaNhaB.

In the present work, we performed site-directed mutagenesis on Asp146 and Asp404 of KpNhaB and determined the consequences of mutating these residues to either Glu or Ala using solid-supported membrane (SSM)-based electrophysiology as main investigation technique. We found that the Glu mutants kept most of the functional characteristics of the WT exchanger. More profound changes, including a change in the transporter’s stoichiometry, were observed when Asp146 was mutated to Ala, either by itself, or together with the D404A mutation. Overall, we found that Asp146 and Asp404 are part of the substrate binding site(s) of KpNhaB and show what is, to our knowledge, the first Na^+^/H^+^ exchanger with a variable stoichiometry.

## Results

### Expression of mutant variants in *E. coli*

**Figure 1:**
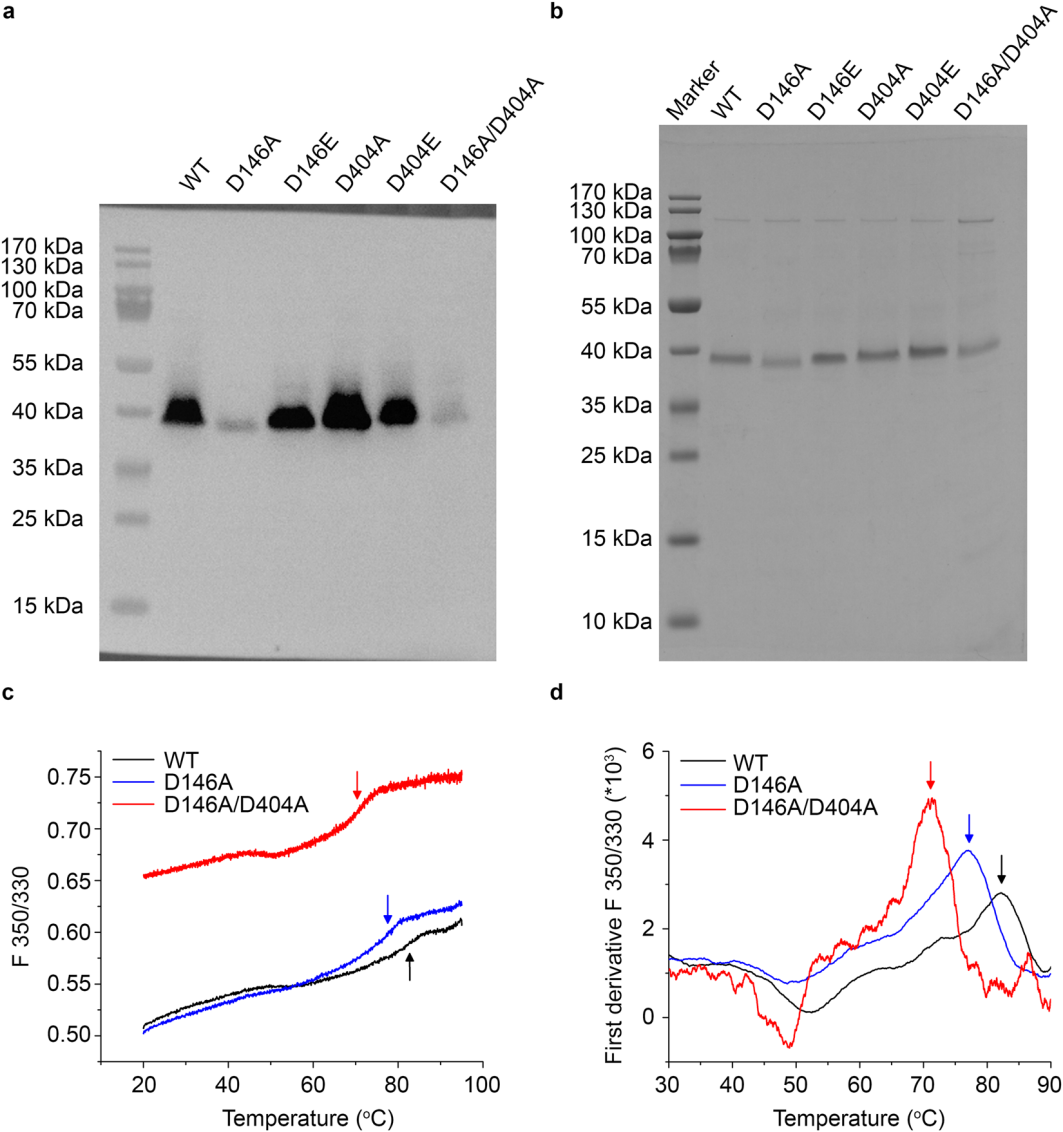
Expression and thermal stability of KpNhaB mutants. (**a**) Western blot of *E. coli* membrane fragments overexpressing KpNhaB variants using an anti-His primary antibody. (**b**) SDS-PAGE of purified KpNhaB variants reconstituted in proteoliposomes visualized using Coomassie blue staining. (**c**) Sample melting curves of KpNhaB WT, D146A and D146A/D404A recorded using DSF. (**d**) First derivative analysis of traces in panel c. Arrows in (**c,d**) indicate the inflection point (T_m_) of each curve. Images in (**a,b)** were acquired using a Fusion FX imaging system and correspond to two independently run gels. The image in (**a**) is an overlay performed by the imaging software of a chemiluminescence image (for the His-tagged protein bands) and a white light image (for the membrane including protein marker positions). The image in (**b**) was obtained using the white light mode of the imaging software. Melting curves in (**c**) are representative results of at least three independent experiments.

KpNhaB was subjected to site-directed mutagenesis and mutant variants were overexpressed in *E. coli* BL21(DE3). Subsequently, the expression level of the mutants was assessed by collecting *E. coli* membranes and subjecting them to SDS-PAGE followed by Western blot using anti-His IgG as primary antibody (Fig. 1a). With the exception of the constructs containing the D146A mutation, most mutants were well expressed at levels comparable to the WT. For the KpNhaB D146A and KpNhaB D146A/D404A mutants, the expression was considerably lower, indicating that the D146A mutation might have a deleterious effect to the stability of the mutant protein. Nevertheless, sufficient amounts (>0.4 mg purified protein) of the D146A and D146A/D404A mutant proteins could be purified. Further, purified proteins were reconstituted in proteoliposomes for further functional assays. Proteoliposomes contained comparable amounts of protein, as shown by SDS-PAGE (Fig. 1b).

### Thermal stability of KpNhaB variants

As the reduced expression level of KpNhaB D146A and D146A/D404A suggested a decreased stability of some KpNhaB mutants, we employed differential scanning fluorimetry to measure the thermal stability of all KpNhaB variants used in our study (Fig. 1c,d and Table 1). A relatively stable protein must be characterized by a melting curve with a well-defined step function and an inflection point (melting temperature, T_m_) at high temperatures^27^. Such is the case for the KpNhaB WT protein (Fig. 1c), which undergoes a major unfolding event that starts at ~60 °C. Using first derivative analysis (Fig. 1d), we could determine the T_m_ to be 82.9 ± 0.3 °C, which is comparable to that obtained for a Na^+^/H^+^ antiporter from a thermophilic organism^28^.

**Table 1:** Properties of KpNhaB variants investigated.

Variant	T_m_ (°C)	I_max_ (nA)	pH_opt_	K_m_ (pH_1_)	K_m_ (pH_2_)	K_m_ (pH_3_)
WT	82.9 ± 0.3	37 ± 1	8.0	74 ± 8 mM, *n* = 2 (7.0)	7 ± 1 mM (8.5)	—
D146E	77.4 ± 0.8	13 ± 3	8.5	170 ± 38 mM (7.0)	20 ± 3 mM (8.5)	—
D404E	76.5 ± 1.2	28 ± 10	8.0	100 ± 9 mM, *n* = 1.2 (7.5)	11 ± 0.1 mM (8.5)	—
D146A	77.6 ± 0.4	4.1 ± 1.4	7.0	37 ± 3 mM (7.5)	4 ± 1 mM (8.5)	2 ± 0.3 mM (9)^*^ 288 ± 59 mM (9)^****^
D404A	75.8 ± 0.9	13 ± 1	8.5	285 ± 19 mM, *n* = 1.2 (7.5)	193 ± 26 mM (8.5)	—
D146A/D404A	70.4 ± 1.1	1.7 ± 0.3^**^ 2.3 ± 0.7^***^	7.0* ≥ 9.5^****^	19 ± 2 mM (7.5)	252 ± 3 mM (8.5)^****^	126 ± 17 mM (9.5)

T_m_ represents the melting temperature determined by DSF as the inflection point for the major unfolding event in the melting curves shown in Fig. 1c (maximum of the first derivative in Fig. 1d). I_max_ represents the maximum value of the peak currents recorded, which is a measure of transporter turnover. pH_opt_ represents the pH conditions were the highest peak currents were recorded. K_m_ (pH) values are apparent affinities determined for the Na^+^ substrate under different pH conditions. Na^+^ affinity values for KpNhaB WT were determined previously^25^. *n* denotes the Hill coefficient of cooperativity where data where fitted with a Hill function. Data shown are average of at least three independent measurements ± s.d.

^*^When only the negative component of the recorded transient currents was considered.

^**^For negative currents, H^+^:Na^+^ > 1.

^***^For positive currents, H^+^:Na^+^ < 1.

^****^When only the positive component of the recorded transient currents was considered.

The melting temperatures determined for the KpNhaB mutants (Table 1) are, without exception, lower than the melting temperature of the WT, indicating that the Asp146 and Asp404 residues play a role in the stability of the exchanger. It has been previously demonstrated that mutations on residues located in the active site of bacterial CPA2 Na^+^/H^+^ antiporters (Lys300 and Asp163 in EcNhaA) lead to systematic reductions in the T_m_ ^29^.

Although mutations on Asp404 seem to affect more the stability than replacements of Asp146, the polarity of the replacing side chain had no effect on the stability, as there was no significant difference between the T_m_ values of the Glu or Ala substitution of the same Asp residue. Given that mutants with a similar T_m_ were shown to have highly different expression levels (Fig. 1a and Table 1), it is likely that the reduced expression of the D146A and D146A/D404A mutants does not directly relate to their thermal stability.

### Electrophysiological characterization of KpNhaB D146E, D404E and D404A

Proteoliposomes of all prepared KpNhaB mutant variants were subjected to SSM-based electrophysiology in order to ascertain the effect of the mutations on the transport function. An electrophysiological characterization of the transport function has already been performed for KpNhaB WT^25^.

SSM-based electrophysiology^24,30^ uses proteoliposomes containing the transporter of interest adsorbed to a hybrid bilayer deposited on a gold electrode. The technique allows the recording of electrical currents generated by transporter activity in response to rapid substrate concentration jumps. The information extracted from SSM-based electrophysiological recordings derives from the (1) polarity of the recorded currents, (2) their maximum amplitude, as well as (3) their decay time, as detailed below:

1. The polarity of the recorded currents indicates the direction in which net charge is transferred. In the context of the SSM-based electrophysiological recordings presented here, a negative current indicates the transfer of net negative charge towards the inside of the proteoliposomes (or net positive charge towards the outside), while a positive current indicates the transfer of net positive charge towards the inside of the proteoliposomes (or net negative charge towards the outside);
2. The maximum amplitude of the recorded currents (“peak current”) is a measure of transporter activity^31,32^;
3. Two types of currents could be recorded throughout the electrophysiological measurements. For clarity, we will call “pre-steady-state” those currents with a fast decay time, which is typically independent of substrate concentration. In our experimental setup, these typically reach the baseline after less than 100 ms. We refer to recorded currents as “steady-state” if they fulfil the following conditions, as discussed previously^29^: (a) they decay slower than pre-steady-state currents, (b) their decay time constants strongly decrease with rising substrate concentration. Note that, due to the capacitively coupled membrane system of the SSM, all recorded currents are transient currents, as previously described^24,32^.

Measurements presented in this work relied on concentration jumps of one of the substrates, Na^+^, from a solution containing no Na^+^ to solutions containing different concentrations of Na^+^. Unless otherwise stated, measurements were performed at symmetrical pH (the same pH inside and outside the proteoliposomes). Under this type of experimental protocol, measurements previously performed on KpNhaB WT^25^ yielded negative currents, in line with a H^+^:Na^+^ stoichiometry >1.

**Figure 2:**
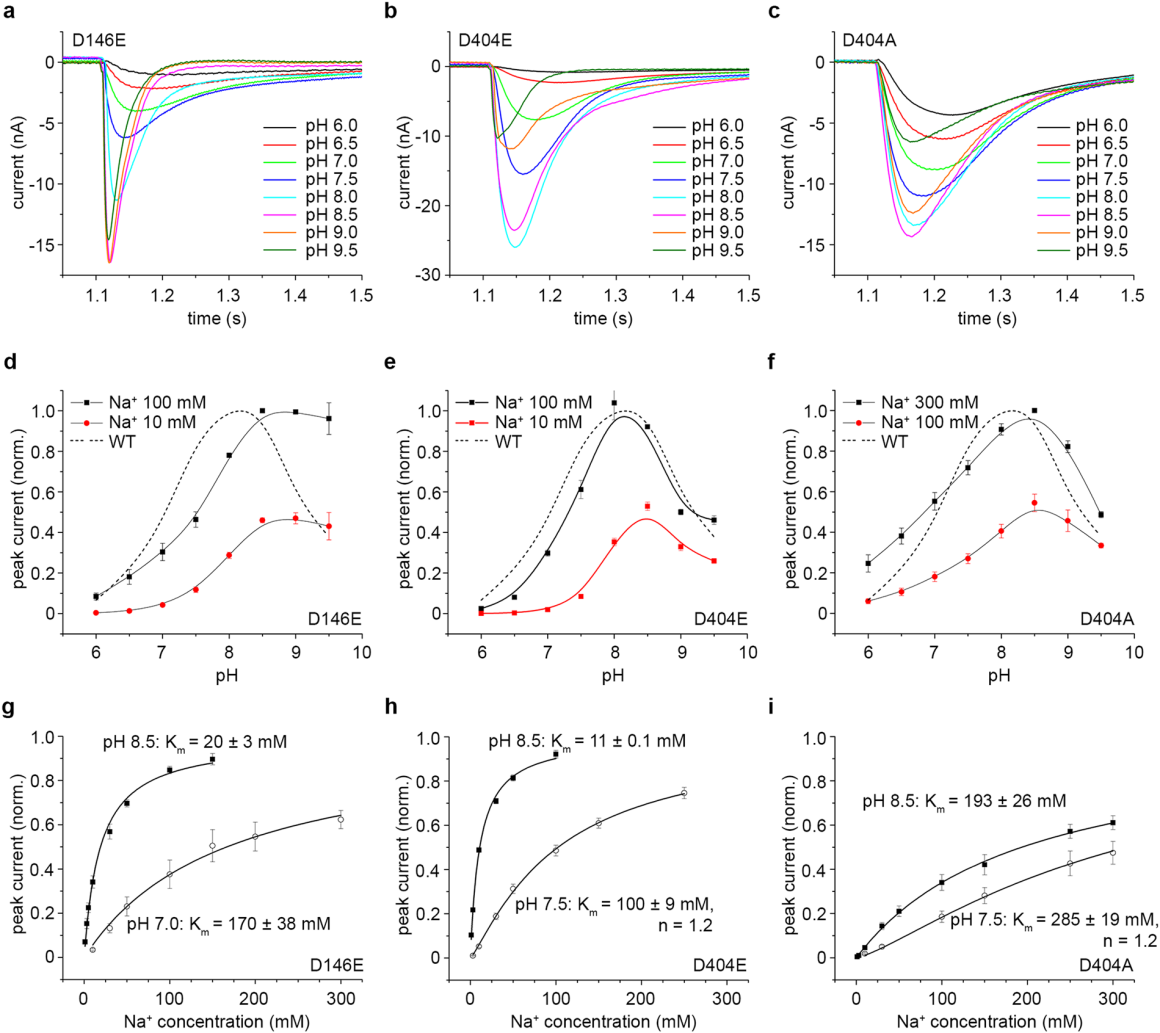
Electrophysiological characterization of KpNhaB D146E, D404E and D404A. (**a–c**) Transient currents recorded for KpNhaB D146E (**a**), D404E (**b**) and D404A (**c**) following Na^+^ concentration jumps of 100 mM (**a,b**) or 300 mM (**c**) under different pH conditions. (**d–f)** pH dependence of the transient current amplitude recorded for KpNhaB D146E (**d**), D404E **(e**) and D404A (**f**). Data were normalized to the maximum determined amplitude. Lines are guides to the eye. The dotted line shows the pH profile of the KpNhaB WT, as determined previously^25^. (**g–i**) Na^+^ concentration dependence of the transient current amplitude recorded for KpNhaB D146E (**g**), D404E (**h**) and D404A (**i**). Data were normalized to the extrapolated maximum of the hyperbolic/sigmoidal fits. Data in (**d–i**) are presented as average of three independent recordings ± s.d.

The KpNhaB D146E and D404E mutants showed most of the characteristics of the WT protein (Fig. 2). Thus, the two mutants displayed a pH-dependent response (Fig. 2a,b), with steady-state currents recorded under most of the pH range investigated. At alkaline pH, (>8 for D146E and >8.5 for D404E), the currents became narrower, indicating mostly a pre-steady-state component, as was the case for KpNhaB WT at pH 9 and 9.5^25^. For D404E, the maximum recorded currents were roughly similar to the WT, while the maximum activity of the D146E mutant was ~ 3 times lower (Table 1).

When comparing their pH-dependent activity profiles (Fig. 2d,e), D404E was remarkably similar to the WT, while D146E displayed no decrease in the amplitude of the transient currents above pH 8.5. The Na^+^ affinity of D404E was comparable to that of the WT at pH 8.5, while that of D146E was roughly threefold lower (Fig. 2g,h and Table 1). At neutral pH, the affinity for Na^+^ decreased in both mutants, consistent with competition between the Na^+^ and H^+^ substrates (Fig. 2g,h and Table 1).

Given that, in most respects, the function of the D404E mutant is similar to KpNhaB WT, we can conclude that Glu can serve as a good replacement for that residue. However, Asp146 seemed to be more sensitive to the exchange to Glu, having a partially compromised transport activity. By comparison, the binding site of EcNhaA seems to be far more sensitive to similar mutations – while replacement of Asp164 with Glu preserves partially the function of the exchanger, mutation of Asp163 to Glu abolishes completely the transport activity^33^.

Replacement of Asp404 with Ala had a more profound effect on the transport activity of the exchanger. Thus, the recorded currents (Fig. 2c) all correspond to steady-state transport, including at alkaline pH. The maximum recorded currents (Table 1) were threefold lower than in the WT, as for the D146E mutant. The pH-dependent activity profile of the mutant is broader (Fig. 2f), with a slower decrease of activity in the acidic range than that of the WT, and with a pH optimum slightly shifted towards the alkaline. The Na^+^ affinity of KpNhaB D404A is reduced more than 20-fold at alkaline pH compared to the WT (Fig. 2i and Table 1), although the difference at neutral pH is only ~ 4-fold. As in the D404E mutant, cooperativity exists at pH 7.5 (Fig. 2i), but with a small Hill coefficient (1.2). By comparison, the Na^+^-dependent activity profile of the KpNhaB WT was strongly cooperative at pH 7, with a Hill constant of 2^25^. As the value of the Hill coefficient is a measure of the interaction energy between the binding sites^34^, a reduced value can be interpreted as a disruption in the interaction between the substrate binding sites of KpNhaB.

### Electrophysiological characterization of KpNhaB D146A

**Figure 3:**
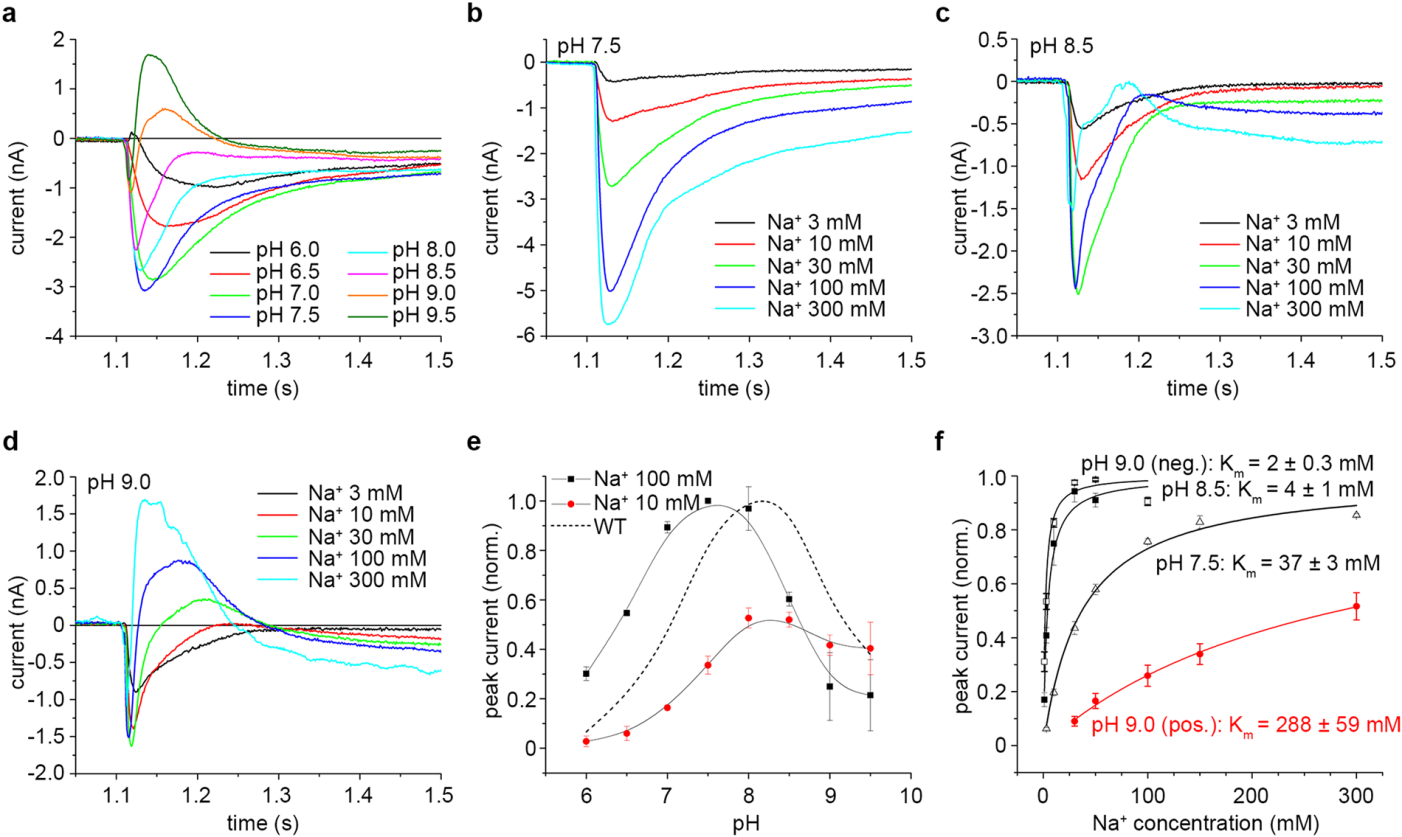
Electrophysiological characterization of KpNhaB D146A. (**a**) Transient currents recorded for KpNhaB D146A following Na^+^ concentration jumps of 100 mM under different pH conditions. (**b**–**d**) Transient currents recorded for KpNhaB D146A following different Na^+^ concentration jumps at pH 7.5 (**b**), pH 8.5 (**c**) and pH 9 (**d**). (**e**) pH dependence of the transient current negative amplitude recorded for KpNhaB D146A. Data were normalized to the maximum determined amplitude. Lines are guides to the eye. The dotted line shows the pH profile of the KpNhaB WT, as determined previously^25^. (**f**) Na^+^ concentration dependence of the transient current amplitude recorded for KpNhaB D146A. Black symbols and lines were used for the data derived from negative peak currents, while red was used for data derived from the positive peak currents at pH 9. Data were normalized to the extrapolated maximum of the hyperbolic fits. Data in (**e,f)** are presented as average of three independent recordings ± s.d.

By comparison to the mutants described above, the effects of the D146A mutation were far more drastic. First, the maximum activity of the mutant exchanger was reduced by about 9 times compared to the WT (Table 1). Second, the shape of the recorded transient currents (Fig. 3a) was markedly different to both the WT and the previously described mutants. Thus, the transient currents recorded at pH 9 and 9.5 for a Na^+^ concentration jump of 100 mM have a major, positive component, that has a fast decay characteristic of a pre-steady-state charge displacement^35^.

A more detailed analysis revealed that the appearance of a second component in the shape of the transient currents was both pH- and Na^+^ concentration-dependent. Whereas transient currents recorded for different Na^+^ concentration jumps at pH 7.5 (Fig. 3b) were all consistent with steady-state electrogenic transport (at a H^+^:Na^+^ stoichiometry >1), at pH 8.5 (Fig. 3c), currents mostly had the characteristics of pre-steady-state charge displacement. At this pH, currents recorded following Na^+^ concentration jumps of 100 mM and higher additionally contained a second, negative, component, which was more pronounced at higher Na^+^ concentrations. Most likely due to the appearance of this second component, the amplitude of the current recorded following a 300 mM Na^+^ concentration jump was markedly smaller than that of currents recorded for 30 or 100 mM Na^+^ concentration jumps at the same pH (Fig. 3c).

When pH was increased to 9, a similar Na^+^-dependent variation in the shape of the transient currents was apparent (Fig. 3d). In this case, the currents recorded for Na^+^ concentration jumps of 3 and 10 mM consisted of a negative, fast component. For Na^+^ concentration jumps of higher concentration, the transient currents started to present a second, positive component, which increased with the Na^+^ concentration.

When the overall pH-dependent activity of the exchanger is considered (Fig. 3e), it is clear that the KpNhaB D146A mutant is acid-shifted relative to the WT by ~0.5 pH units. Overall, the affinity for Na^+^ of the exchanger (Fig. 3f and Table 1) is comparable to that of the WT at pH 8.5, decreasing ~9 fold at pH 7.5. At pH 9, analysis of the negative component of the transient currents revealed an affinity for Na^+^ of 2 mM (Table 1), only slightly increased from the 4 mM at pH 8.5. The positive component of the transient currents also increased hyperbolically with the Na^+^ concentration, with a K_m_ of ~290 mM (Table 1).

### Electrophysiological characterization of KpNhaB D146A/D404A

**Figure 4:**
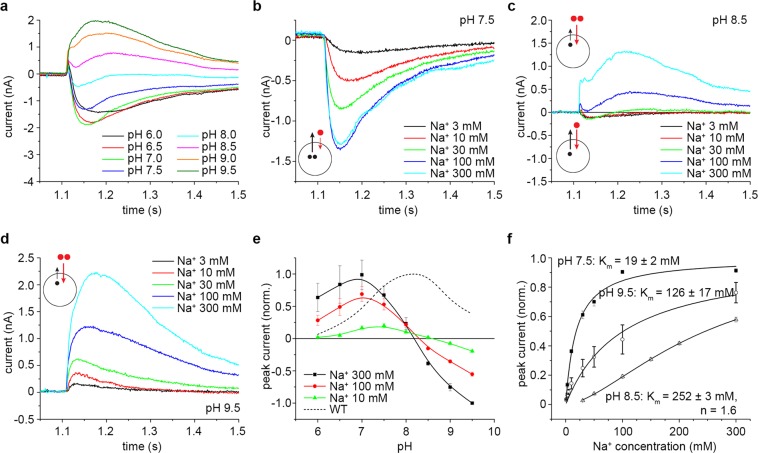
Electrophysiological characterization of KpNhaB D146A/D404A. (**a**) Transient currents recorded for KpNhaB D146A/D404A following Na^+^ concentration jumps of 300 mM under different pH conditions. (**b**–**d**) Transient currents recorded for KpNhaB D146A/D404A following different Na^+^ concentration jumps at pH 7.5 (**b**), pH 8.5 (**c**) and pH 9.5 (**d**). Insets show the variable transport stoichiometries exhibited under different conditions, with black circles denoting H^+^ and red circles denoting Na^+^. At pH 8.5 (**c**), two modes are possible depending on the Na^+^ concentration employed. See the Discussion for more details. (**e**) pH dependence of the transient current amplitude recorded for KpNhaB D146A/D404A. Data were normalized to the peak current recorded at pH 9.5 for a concentration jump of 300 mM Na^+^. Positive currents correspond to the mutant working according to a H^+^:Na^+^ > 1 stoichiometry (similar to the WT), while negative currents correspond to the mutant working according to a H^+^:Na^+^ < 1 stoichiometry. Lines are guides to the eye. The dotted line shows the pH profile of the KpNhaB WT, as determined previously^25^. (**f**) Na^+^ concentration dependence of the transient current amplitude recorded for KpNhaB D146A/D404A. Data were normalized to the extrapolated maximum of the hyperbolic/sigmoidal fits. Data in (**e,f**) are presented as average of three independent recordings ± s.d.

As the behavior of both the D146A and the D404A mutants indicated that mutation of either of these two aspartates to alanine induced profound changes in the transport activity of KpNhaB, we constructed the double mutant D146A/D404A. Suprisingly, in KpNhaB D146A/D404A, slow, negative currents corresponding to steady-state transport at a H^+^:Na^+^ stoichiometry >1 could only be recorded below pH 8 (Fig. 4a). At pH 8.5 and above, however, currents retained the characteristics of steady-state transport, but reversed in polarity, showing steady-state transport at a H^+^:Na^+^ stoichiometry <1.

Indeed, when looking at the Na^+^ dependence of the recorded transients at different pH values (Fig. 4b–d), the same tendency could be observed. At pH 7.5 (Fig. 4b) all currents were negative and slow, showing steady-state transport of negative charge towards the interior of the proteoliposomes (H^+^:Na^+^ > 1). At pH 8.5 (Fig. 4c) and low Na^+^ concentration only a small, fast transient consistent with a pre-steady-state charge displacement was apparent. Above 30 mM Na^+^, a slow, positive component appeared in the transients, indicating steady-state transport of positive charge towards the interior of the proteoliposomes (H^+^:Na^+^ < 1). Finally, at pH 9.5 (Fig. 4d) all currents were positive, showing steady-state transport consistent with a H^+^:Na^+^ stoichiometry <1.

This behavior can be well visualized in Fig. 4e, where the pH dependence of the recorded transients is shown for three different Na^+^ concentrations. The positive direction in Fig. 4e corresponds to the “normal” operating mode of KpNhaB (H^+^:Na^+^ > 1, negative currents), which appears in the pH range 6–8. At pH 8.5, currents reverse polarity and increase with pH, reaching at pH 9.5 maxima similar to those recorded at pH 7, only of opposite sign (Fig. 4e and Table 1).

When the affinity for Na^+^ was determined (Fig. 4f and Table 1), another anomalous behavior compared to the other mutants was recorded. Whereas, in other variants, the apparent K_m_ values decreased with the increase of pH, the determined affinities for KpNhaB D146A/D404A are 19 mM at pH 7.5 and 252 mM at pH 8.5. This is followed by a subsequent decrease at pH 9.5 to 126 mM.

### Electrophysiological recordings under asymmetrical pH conditions

As the behavior of the D146A, and especially the D146A/D404A mutants was anomalous, and, in the case of the latter, at least, consistent with a drastic change of stoichiometry, we also performed SSM-based electrophysiological measurements under conditions of asymmetrical pH. Unlike the experiments shown in Figs 2–4, where the pH was the same on either side of the proteoliposomal membrane, in the asymmetrical pH experiments the interior of the proteoliposomes was acidic (pH_in_ = 6), ensuring adequate protonation of the transporter, while the outside (where Na^+^ was bound) was at high pH (pH_out_ = 9.5).

**Figure 5:**
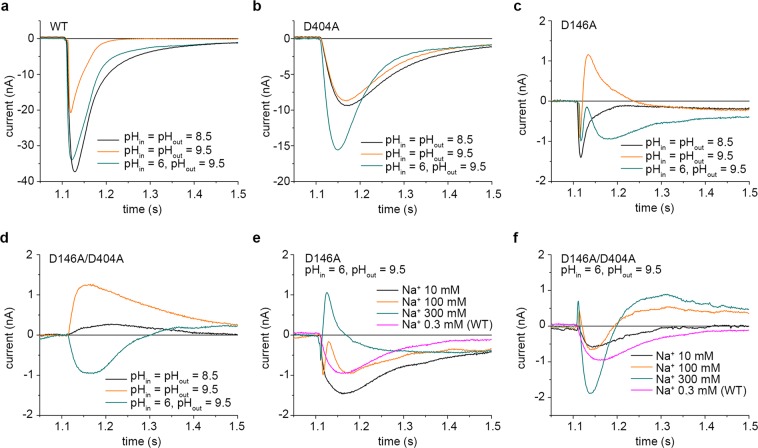
Electrophysiological measurements under asymmetrical pH conditions across the proteoliposome membrane. (**a–d**) Transient currents recorded for Na^+^ concentration jumps of 100 mM using either the same pH inside and outside the proteoliposomes (pH_in_ = pH_out_) or pH 6 inside (pH_in_) and 9.5 outside the proteoliposomes (pH_out_). Transient currents were recorded for KpNhaB WT (**a**), KpNhaB D404A (**b**), KpNhaB D146A (**c**) and KpNhaB D146A/D404A (**d**). (**e,f**) Transient currents recorded under conditions of asymmetrical pH (pH_in_ = 6, pH_out_ = 9.5) recorded at different Na^+^ concentrations for KpNhaB D146A (**e**) and KpNhaB D146A/D404A (**f**). In panels (**e**) and (**f**), a Na^+^ concentration jump of 0.3 mM recorded for KpNhaB WT under the same conditions is shown for reference.

It can be seen (Fig. 5a) that KpNhaB WT behaved as expected under these conditions. Thus, under symmetrical pH conditions, the recorded current at pH 8.5 was a slow one, corresponding to steady-state turnover, while at pH 9.5 it was a fast, pre-steady-state signal, as previously shown^25^. The behavior at pH 9.5 is explained by insufficient protonation, which does not allow the full transport cycle to proceed, whereas the recorded signal is a pre-steady-state transient corresponding to Na^+^ binding^31,36^. At asymmetrical pH (pH_in_ = 6, pH_out_ = 9.5), when the protonation of the exchanger is ensured by the inside acidic pH, the recorded current is virtually identical to the one at symmetrical pH 8.5.

In the case of D404A, the transient currents recorded for 100 mM Na^+^ concentration jumps under symmetrical pH were roughly similar at pH 8.5 and pH 9.5 (Fig. 5b). The recorded current increased slightly when the inside pH was acidic, indicating that additional protonation favored a more rapid turnover. Steady-state turnover was apparent under all employed conditions.

Surprinsingly, KpNhaB D146A showed steady-state turnover under asymmetrical pH conditions (pH_in_ = 6, pH_out_ = 9.5), despite showing only pre-steady-state charge translocation under both symmetrical pH conditions tested, pH 8.5 and 9.5 (Fig. 5c). This is consistent with a H^+^:Na^+^ stoichiometry >1 when the inside pH was acidic. For the double mutant D146A/D404A (Fig. 5d), the shape of the transient current recorded under asymmetrical pH conditions suggests a pre-steady-state charge displacement, indicating a 1:1 H^+^:Na^+^ stoichiometry.

When Na^+^ concentration jumps of different values were performed under asymmetrical pH conditions (pH_in_ = 6, pH_out_ = 9.5), the nature of the transient currents was found to be Na^+^-dependent. Thus, for both the D146A (Fig. 5e) and D146A/D404A (Fig. 5f) mutants, the currents recorded at low Na^+^ concentration were slow, indicating electrogenic steady-state turnover. As the Na^+^ concentration was increased, currents became faster, showing only pre-steady-state charge displacement.

### Fluorimetric measurements of membrane potential

**Figure 6:**
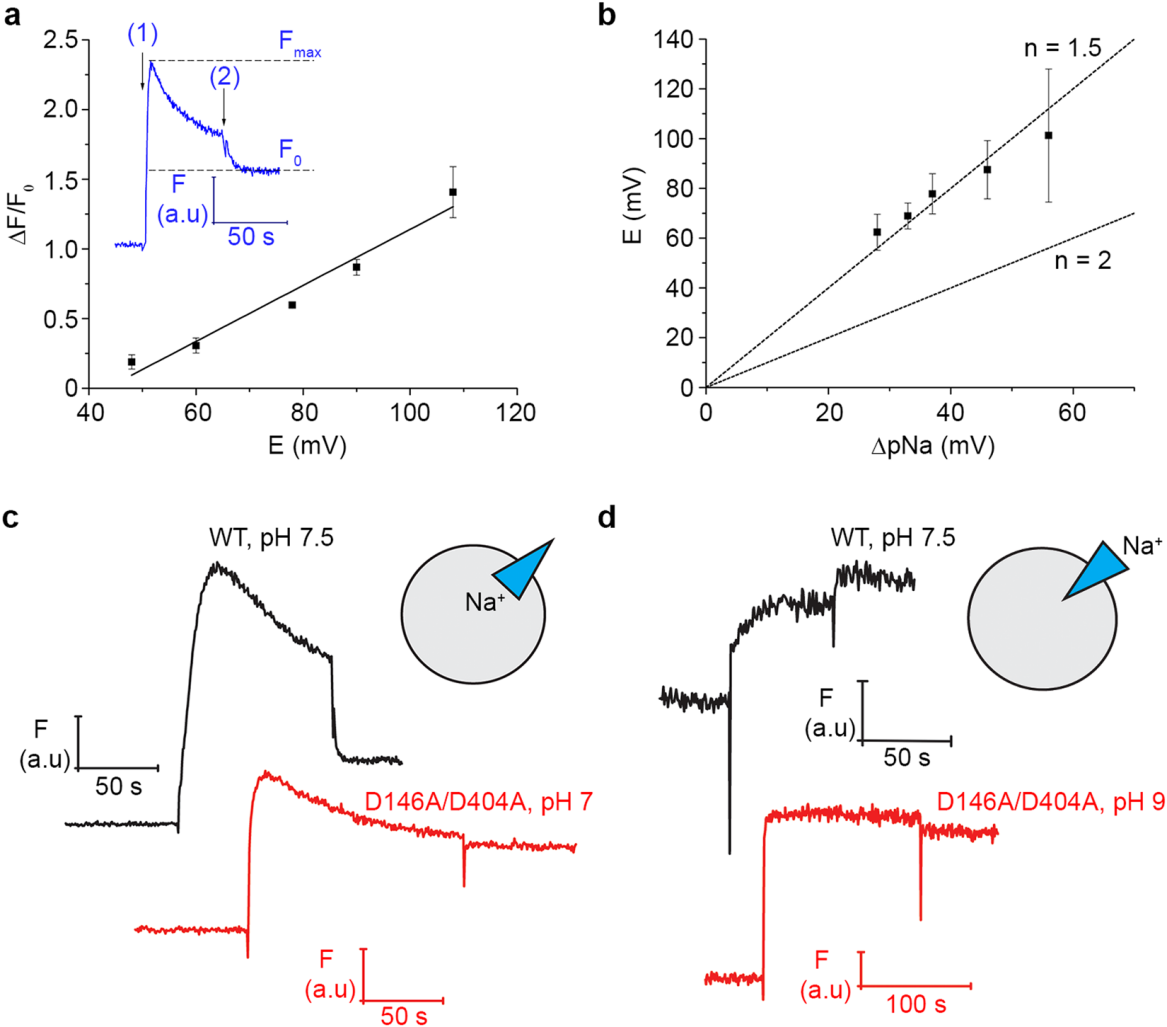
Fluorimetric measurements of membrane potential. (**a**) Calibration of oxonol VI fluorescence using potentials generated by K^+^ gradients in the presence of valinomycin. Inset shows a sample fluorescence curve, where (1) represents addition of liposomes to the fluorescence cuvette and (2) the addition of uncoupler in order to obtain baseline fluorescence. The uncoupler concentrations used were 1 μM monensin for measurements on empty liposomes (**a**) and 1 μM SF6847 for measurements on proteoliposomes (**b–d**). (**b**) Determination of KpNhaB WT stoichiometry at pH 7.5. The two lines represent theoretical curves for a H^+^:Na^+^ stoichiometry of 2:1 (n = 2) or 3:2 (n = 1.5). (**c**) Potentials generated by KpNhaB WT and D146A/D404A in presence of an outward-directed sodium concentration gradient ([Na^+^]_in_ > [Na^+^]_out_). (**d)** Potentials generated by KpNhaB WT and D146A/D404A in presence of an inward-directed sodium concentration gradient ([Na^+^]_in_ < [Na^+]^_out_). Data in (**a,b**) are averages of 3–5 independent recordings ± s.d. Traces in (**c**,**d**) are representative of at least three independent recordings.

Finally, to corroborate our electrophysiological findings, we employed the potential-sensitive dye oxonol VI in order to detect and quantify the formation of inside-positive potentials in liposomes. The response of the dye was calibrated using liposomes loaded with 0.5 mM K^+^ which were diluted in solutions containing variable amounts of K^+^ in the presence of valinomycin, allowing the rapid establishment of a membrane potential detected as an increase in the fluorescence of the oxonol dye (Fig. 6a). The calibration over the range 48–108 mV yielded essentially a linear response (Fig. 6b).

Figure 6b shows the determination of KpNhaB WT stoichiometry using proteoliposomes at a lipid to protein ratio of 100. Theoretical curves corresponding to a stoichiometry of 1.5 or 2 were plotted, and it can be seen that the results obtained fit a stoichiometry of 1.5, which is the same as that described for the *E. coli* NhaB exchanger^18^.

When KpNhaB D146A/D404A proteoliposomes were used, we were not able to detect formation of a membrane potential at high lipid to protein ratio values (data not shown). This is consistent with the up to 20 times lower turnover of the mutant compared to the WT (Table 1). When proteoliposomes of the mutant at a lipid to protein ratio of 5 were used at pH 7, formation of a positive-inside membrane potential could be detected (Fig. 6c), although the amplitude of the fluorescence change was highly reduced in comparison to the WT. As such, we decided not to use the obtained potential values for quantitative determinations of stoichiometry.

Under conditions where proteoliposomes were loaded with low amounts of Na^+^ and diluted in high concentration Na^+^ solutions, KpNhaB WT is expected to generate a negative-inside membrane potential, due to its H^+^:Na^+^ stoichiometry >1. Indeed, KpNhaB WT proteoliposomes at a lipid to protein ratio of 100 generated, under these conditions, a lower than baseline level of oxonol VI fluorescence (Fig. 6d). This is in line with exclusion of the anionic dye from the interior of negatively-charged proteoliposomes. When the same measurement was repeated at pH 9 using KpNhaB D146A/D404A proteoliposomes at a lipid to protein ratio of 5, a positive potential was generated instead (Fig. 6d), which confirms our electrophysiological findings that, under those conditions, the mutant operates at a H^+^:Na^+^ stoichiometry <1.

## Discussion

### Aspartate residues in Na^+^/H^+^ exchange

Analysis of solved protein structures containing sodium binding sites reveals a clear preference of the sodium ion to be coordinated by oxygen atoms, belonging to either the main chain or secondary chain of amino acid residues, typically with a coordination number of 6^37–39^. While there seems to be no *a priori* requirement to have a negatively charged secondary chain in order for an amino acid residue to coordinate sodium in a typical binding site^38^, a special consideration has to be given for the binding sites of Na^+^/H^+^ exchangers. Experimental evidence collected on several prokaryotic Na^+^/H^+^ exchangers, including KpNhaB, shows that the transport mechanism of Na^+^/H^+^ exchangers is explained by competition of Na^+^ and H^+^ for a common binding site^26,31,35,36^. As such, a common binding site for H^+^ and Na^+^ requires the existence of at least one protonatable residue. In that respect, Asp and Glu residues are the most likely residues to contribute to Na^+^ and H^+^ binding in Na^+^/H^+^ exchangers. Indeed, the single structure of a Na^+^/H^+^ exchanger that has been solved to a resolution that allowed identification of the metal atom (using Tl^+^ instead of Na^+^), that of the NhaP exchanger from *Pyrococcus abyssi*^13^ shows the presence of both an aspartate (Asp159) and a glutamate (Glu73) residue coordinating the metal ion. Furthermore, there is clear evidence to the involvement of Asp residues in Na^+^ binding sites in other Na^+^/H^+^ exchangers, such as Asp163 and Asp164 in EcNhaA^3^ and Asp161 in MjNhaP1^36^.

Given these facts, targeting of Asp146 and Asp404 in KpNhaB for mutation was logical, as, according to the single topological study of an NhaB exchanger^17^, they are the only Asp residues in the putative transmembrane domains of NhaB. Our results clearly show the involvement of these two residues in the KpNhaB substrate binding site(s), as discussed below. When comparing our results with the previous study of the homologous Asp147 of VaNhaB^21^, the superiority of SSM-based electrophysiology over classical fluorescence dequenching experiments performed on membrane vesicles is immediately apparent. Thus, instead of classifying mutants with lower expression levels and lower activity as simply inactive, the use of SSM-based electrophysiology allowed a complete characterization of these mutants.

### Steady-state positive currents in KpNhaB D146A/D404A show a change in stoichiometry

As previously detailed, the polarity of the recorded currents indicates the direction in which net charge is transferred. Note that, as previously described, currents recorded for steady-state transport are transient due to the capacitively coupled recording technique.

**Figure 7:**
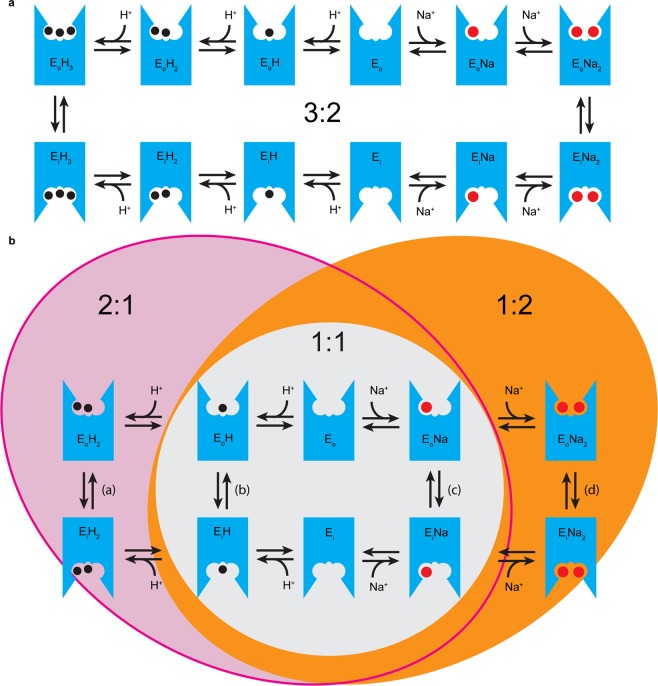
Proposed model of the transport mechanism of KpNhaB WT (**a**) and the D146A/D404A mutant (**b**). The exchanger can switch between its outside open (E_o_) and inside open (E_i_) forms only when bound to Na^+^ (red circle) or H^+^ (black circle). (**a**) The WT can only function at a H^+^:Na^+^ stoichiometry of 3:2. (**b**) KpNhaB D146A/D404A can function at different stoichiometries depending on the conditions employed (pH, Na^+^ concentration). 3 possible stoichiometries are shown, each is a cycle closed by two substrate translocation reactions: a 2:1 mode (a + c), a 1:1 mode (b + c) and a 1:2 mode (b + d). A 2:2 mode (a + d, not pictured explicitly) is also possible for the mutant under asymmetrical pH conditions.

The currents recorded for KpNhaB WT under all conditions were negative^25^. Considering that, in our SSM experiments, the proteoliposomes were exposed to Na^+^ only from the outside, this requires a H^+^:Na^+^ stoichiometry >1^18^. This is confirmed by our direct measurements of stoichiometry using oxonol VI (Fig. 6b) that show KpNhaB WT to have the same stoichiometry as that previously determined for NhaB in *E. coli*, of 3:2H^+^:Na^+^ ^18^. The transport mechanism describing the 3:2 stoichiometry of the WT protein is shown in Fig. 7a.

In KpNhaB D146A/D404A, steady-state negative currents were recorded at pH < 8, just as in the WT. However, when pH was increased, positive, steady-state currents were recorded, indicating that at pH > 8.5, the H^+^:Na^+^ stoichiometry is <1 (Fig. 4a,c,d), a finding confirmed by fluorimetric measurements (Fig. 6d). This, is to our knowledge, the first reported case of a Na^+^/H^+^ exchanger that can operate in such a mode. Furthermore, it represents the first reported case of a Na^+^/H^+^ exchanger with a variable stoichiometry.

### Asp146 carries one of the H^+^ exchanged by KpNhaB

When KpNhaB D146A and D146A/D404A proteoliposomes were subjected to Na^+^ concentration jumps under asymmetrical pH conditions (pH_in_ ≪ pH_out_), the response was Na^+^ concentration-dependent (Fig. 5e,f). At low Na^+^, steady-state negative currents were recorded, showing a H^+^:Na^+^ stoichiometry >1. At higher Na^+^ concentrations, currents became pre-steady-state, indicative of an overall electroneutral transport cycle, and thus a H^+^:Na^+^ stoichiometry of 1.

As apparent from these measurements as well as the behavior of the two mutants under symmetrical pH conditions (Figs 3 and 4), it is clear that the two mutants are still capable of transporting 2 Na^+^ ions at sufficiently high Na^+^ concentration. However, we can only account for the stoichiometry of 1 obtained using high Na^+^ concentrations in the asymmetrical pH measurements if we accept that the mutants can bind at most 2 H^+^ ions, transport occurring under those conditions at a 2:2 H^+^:Na^+^ ratio. As the WT transports 3 H^+^ ions, we can assign Asp146 as the binding site of one of the H^+^ ions transported by KpNhaB.

### The different transport modes of KpNhaB D146A/D404A

Electrophysiological measurements performed on KpNhaB D146A/D404A under symmetrical pH conditions, pH_in_ = pH_out_, allowed the recording of three types of currents (Fig. 4a–d): (1) negative, steady-state (obtained at pH 7.5 and below), (2) negative, pre-steady-state (obtained at pH 8 or pH 8.5 at low Na^+^ concentration) and (3) positive, steady-state (obtained at pH 8.5 at high Na^+^ concentration and at higher pH values). Considering the maximum number of H^+^ transported in the mutant is 2, as discussed above, we can explain the 3 types of currents obtained by three distinct transport modes of the mutant (Fig. 7b) with three different H^+^:Na^+^ stoichiometries, 2:1, 1:1 and 1:2.

Such a variable stoichiometry can only exist if, in the double mutant, the requirement that both Na^+^ (or H^+^) binding sites are fully occupied in order for the transporter to translocate in its substrate-bound form is removed (Fig. 7). For example, while in KpNhaB WT, Na^+^ translocation occurs only when the two Na^+^ binding sites are occupied (ENa_2_ form), the mutant exchanger becomes partially uncoupled – it can translocate Na^+^ over the membrane in two possible conformations (Fig. 7), depending on the amount of Na^+^ available – loaded with one Na^+^ (ENa, at low Na^+^) or with two Na^+^ (ENa_2_, at high Na^+^ concentrations). Similarly, H^+^ transport is also partially uncoupled and the double mutant can transport H^+^ over the membrane in either the EH or the EH_2_ form (Fig. 7b), whereas the WT (Fig. 7a) can only perform H^+^ translocation when it is fully protonated (EH_3_ form).

Thus, we can assign the recorded currents for the double mutant as follows (Supplementary Fig. S1). At pH values lower than 8, electrogenic, steady-state transport occurs at a 2:1 H^+^:Na^+^ ratio. A second Na^+^ ion cannot bind under these conditions due to it being outcompeted by the high number of H^+^ available. At higher pH values, positive steady-state currents are recorded when transport occurs at a H^+^:Na^+^ ratio <1. Thus, under those conditions, only a single H^+^ can be transported, at a stoichiometry of 1:2 H^+^:Na^+^. At pH 8.5 (Fig. 4c), a mixed type of behavior can be observed – currents recorded for concentration jumps at high Na^+^ concentrations are positive, steady-state, corresponding to the 1:2 H^+^:Na^+^ stoichiometry observed at higher pH values. Currents recorded for concentration jumps at low Na^+^ are, instead, negative, pre-steady-state, indicating a 1:1 H^+^:Na^+^ stoichiometry.

Finally, in the experiment performed using a pH gradient (pH_in_ = 6, pH_out_ = 9.5), the exchanger can operate in two modes depending on the available Na^+^ (Fig. 5f and Supplementary Fig. S1): at low Na^+^, it performs steady-state, electrogenic exchange at a 2:1 H^+^:Na^+^ ratio, while at high Na^+^ it performs electroneutral exchange at a 2:2 ratio and only a pre-steady-state transient current can be recorded.

### Two Na^+^ binding/translocation steps can be identified in KpNhaB D146A

Whereas, in KpNhaB D146A/D404A, steady-state positive currents were recorded at alkaline pH, the currents recorded for the D146A mutant under the same conditions have a more complex shape, with a positive component that we assigned to a pre-steady-state charge displacement. In the framework of the competition model of Na^+^/H^+^ exchange^26^, which was shown to apply to KpNhaB WT as well^25^, the existence of a pre-steady-state current at alkaline pH was explained by the isolation of the Na^+^ binding/translocation reaction under conditions where the small concentration of H^+^ available prevents steady-state turnover^31,36^. The pre-steady-state currents recorded for D146A at alkaline pH can thus be assigned to Na^+^-dependent reactions in the transport cycle. As they are strongly electrogenic processes, it is unlikely that they represent only Na^+^ binding to the substrate binding site(s), but rather also include the subsequent steps of substrate occlusion and/or translocation. For the sake of simplicity, we refer to these events as Na^+^ translocation.

As with the D146A/D404A mutant (Fig. 7b), there are two possible Na^+^ translocation reactions, which can then account for the two polarities of pre-steady-state currents recorded at high pH: the negative component corresponds to Na^+^ translocation when the exchanger is in the ENa form, while the positive component corresponds to Na^+^ translocation in the ENa_2_ form. It is then clear that the mutant possesses two Na^+^ binding sites with distinct affinities, which can then be assigned the two K_m_ values determined at pH 9 for the two polarities of pre-steady-state currents recorded (Table 1): 2 mM for the first, high affinity binding site and 288 mM for the second, low affinity one. The large difference between the affinities of the binding sites also explains why the currents recorded at high pH contain both a positive and a negative component – with the Na^+^ concentrations used in the experiment of up to 300 mM, the low affinity Na^+^ binding site of the mutant is insufficiently saturated and the currents correspond to Na^+^ translocation when only part of the population of exchanger molecules is in the ENa_2_ form, while another part is in the ENa form.

### Effects of the D404A mutation in KpNhaB

The most obvious consequence of the D404A mutation was found to be a severe reduction in the affinity of the exchanger for Na^+^. Thus, at alkaline pH, a more than twentyfold reduction in the Na^+^ affinity was observed in the D404A mutant compared to the WT (Fig. 3i and Table 1). Another result of the D404A mutation was that all recorded currents in the pH range investigated showed only steady-state transport (Fig. 2c), even at alkaline pH where the WT^25^, D146E and D404E mutants (Fig. 2a,b) show only pre-steady-state transient currents. In KpNhaB WT, the pre-steady-state currents recorded at alkaline pH are negative, indicating the translocation of negative charge over the membrane. The absence of these currents in KpNhaB D404A might be explained by the removal of at least part of this charge caused by the replacement of a negatively charged aspartate residue with a non-polar alanine. However, an alternative explanation for this phenotype is an increased pK value of the mutant compared to the WT. Indeed, a fit of the kinetic model previously proposed for KpNhaB WT^25^ revealed a pK of 8.5 for the D404A variant compared to that of 8 determined for KpNhaB WT.

Why are positive steady-state currents obtained in D146A/D404A, then, but not in the D146A single mutant? When comparing the D146A mutant with the D146A/D404A variant (Figs 3 and 4 and Table 1), we can conclude that the D404A mutation lowered the affinity of the high affinity Na^+^ binding site of D146A. Second, a higher pK in KpNhaB D146A/D404A compared to the D146A mutant would explain why at high pH, the double mutant can transport 1 H^+^ and perform steady-state transport at a H^+^:Na^+^ < 1 ratio, while the single mutant cannot.

### Asp146 and Asp404 are part of the substrate binding site(s) of KpNhaB

Overall, our results show that both Asp146 and Glu404 are involved in substrate binding of KpNhaB. We could assign Asp146 as the binding site of one of the three H^+^ ions transported. Furthermore, mutation of both Asp146 and Glu404 clearly affects Na^+^ binding, making it very likely that these residues are part of the Na^+^ binding site(s) of the exchanger. Taking into account the existence of two Na^+^ binding sites in NhaB proteins, in the absence of structural information, it is not, however, clear whether these two residues are part of the same site or of two distinct ones.

In the absence of both protonatable residues, as is the case of the D146A/D404A mutant, transport function is severely impaired and the stability of the exchanger is starkly reduced. However, the double mutant is still able to perform Na^+^/H^+^ exchange, albeit with a highly altered phenotype. It is, thus, apparent that the presence of these two Asp residues in the substrate site of KpNhaB, while essential for the correct function of the antiporter, is not completely indispensable.

As KpNhaB D146A/D404A is still capable of transporting two H^+^ ions, it is clear that other protonatable residues must exist in the TMs of KpNhaB. What are the identities of these residues? According to the proposed model of the secondary structure of NhaB^17^, likely candidates in KpNhaB are Lys110, Glu319 and Glu494, although the last two residues are not conserved in VaNhaB^25^. Future functional and structural studies will be required to answer this question.

## Methods

### Genetic constructs

The genetic coding sequence for the Na^+^/H^+^ exchanger NhaB from *K. pneumonia*e was synthesized by Genscript (Piscataway, NJ, USA) and cloned into the expression vector pET-21d(+) as described previously^25^. The sequence of the *nhaB* gene used, optimized for expression in *E. coli*, is shown in Supplementary Table S1. Recombinants were designed with a 6-His tag at the C-terminal end of the translated protein. Insertion of the single point mutations in the coding sequence of KpNhaB was performed via site-directed mutagenesis using the recombinant *nhaB-pET21d* as template and oligonucleotides designed to introduce the desired nucleotide change, as shown in Supplementary Table S2. Digestion of the non-mutated parent template was achieved by incubation of the PCR product with DpnI endonuclease. The DNA of each construct was verified by sequencing performed by Eurofins Genomics (Ebersberg, Germany).

### Overexpression, purification and reconstitution

C-terminally His-tagged KpNhaB protein variants were expressed and purified as previously described^40^. Briefly, bacterial cells of *E. coli* strain BL21(DE3) were transformed with the corresponding recombinant and cultured in Luria Bertani medium at 37 °C up to OD_600_ = 0.6–0.8. Protein overexpression was induced by addition of 0.75 mM isopropyl β-D-1-thiogalactopyranoside (IPTG) and the bacterial culture was continued for 3 h. Cells were collected using centrifugation at 10,000 g and were disrupted using a French Press pressure cell. Membrane fragments were collected by centrifugation at 100,000 g. Protein purification was performed in n-dodecyl-ß-D-maltoside (DDM) detergent using immobilized Ni^2+^-affinity chromatography. Reconstitution of the detergent-purified protein variants into liposomes was performed using *E. coli* polar lipids extract (Avanti Polar Lipids, Alabaster, AL, USA) at a lipid to protein ratio of 10, as previously described^31^.

Expression of the mutant variants in the *E. coli* plasma membrane was assessed via SDS-PAGE and Western blot using a mouse anti-His IgG primary antibody (Penta-His, Qiagen, Hilden, Germany). Goat anti-mouse-HRP (Dako Denmark A/S, Glostrup, Denmark) was used as secondary antibody. Pageruler Prestained Protein Ladder was used as a marker (Thermo Fisher Scientific, Waltham, MA, USA). Images were collected by chemiluminescence using a Fusion FX imaging system (Vilber Lourmat, Marne-la-Vallée, France). Quantification of purified protein in proteoliposomes was performed using SDS-PAGE followed by Coomassie blue staining. Images were collected in the white light mode of the Fusion FX imaging system.

### Differential scanning fluorimetry (DSF)

Thermal stability of the purified KpNhaB variants was assessed as described previously^29^. In brief, glass capillaries were loaded with the detergent-solubilized proteins at a concentration of 0.5 mg/mL in buffer containing 100 mM KCl, 5 mM MgCl_2_, 25 mM KCH_3_COO (pH 4) and 0.03% n-dodecyl β-D-maltoside (DDM). The capillaries were placed in the thermal plate of a Prometheus NT.48 instrument (NanoTemper Technologies, Munich, Germany). A temperature ramp between 20 and 95 °C at a change rate of 1 °C/min was applied to the protein samples and the emission fluorescence was continuously followed at 350 and 330 nm upon excitation at 280 nm with a power setting of 10%. Thermal protein unfolding events can be detected thanks to the high sensitivity of tryptophan and tyrosine amino acids to changes in their local microenvironment. Every time these residues are exposed to hydrophilic conditions, as a consequence of structural changes, their quantum yield decreases, which leads to shifts in the wavelength of maximum fluorescence and alterations in fluorescence intensity. To take into account these two effects, the ratio between the emission fluorescence at 350 and 330 nm (F 350/330) was plotted versus the temperature in a melting curve^41^. The temperature at which half of the protein population is unfolded, or melting temperature (T_m_), was determined from the inflection point of every melting curve via first derivative analysis.

### SSM-based electrophysiology

Electrophysiological measurements followed the same principles described elsewhere^32^, but were performed using a SURFE^2^R N1 instrument (Nanion Technologies, Munich, Germany)^30^. Briefly, an alkyl-mercaptan monolayer was formed on top of a 3 mm gold-coated sensor by incubation in an ethanolic solution of 1 mM octadecanethiol. The solid-supported membrane (SSM) was subsequently formed by addition of a diphytanoyl-phosphatidylcholine:octadecylamine mixture (60:1) in n-decane at a lipid concentration of 15 mg/mL. 10 µL of proteoliposomes at a lipid to protein ratio of 10 were added at a 1 mg/mL lipid concentration directly on top of the SSM and the sensor was centrifuged using 2500 g at room temperature for 30 min to promote adsorption.

A rapid single solution exchange protocol was employed in order to generate Na^+^ concentration jumps on the immobilized proteoliposomes under symmetrical conditions of pH (pH_in_ = pH_out_). The solution exchange consisted of three consecutive steps of 1 s each in which two different solutions, non-activating (NA) and activating (A), were exchanged in the sequence NA-A-NA. All solutions contained 25 mM MES, 25 mM HEPES, 25 mM Tris and 5 mM MgCl_2_ titrated to the desired pH with HCl or KOH. In addition, NA solutions contained 300 mM KCl, whereas A solutions contained instead *x* mM NaCl and (300-*x*) mM KCl. Measurements under asymmetrical conditions of pH (pH_in_ ≠ pH_out_) were performed by incubation of the SSM in a resting solution (R) for 20–30 minutes prior to the NA-A-NA solution exchange. The composition of the resting solution was the same as the NA solution but titrated to a different pH, such that the Na^+^ concentration jump was achieved under a pH gradient. In every case, the section of the recorded current traces corresponding to the NA-A solution exchange (ON transient) was taken for analysis. The peak current amplitude of the ON transient was taken as representative of the steady state Na^+^/H^+^ exchange activity, unless otherwise indicated.

### Fluorimetric measurements

The potential-sensitive dye oxonol VI^42^ was used to detect formation of membrane potentials in proteoliposomes and to measure transport stoichiometry, using a protocol derived from previously described determinations^18,43^. Measurements were performed using a Hitachi F4500 Fluorimeter at the wavelengths λ_ex_ = 614 nm and λ_em_ = 646 nm. Calibration of the dye for measurement of positive inside potentials was performed using liposomes prepared from *E. coli* polar lipid extract (Avanti Polar Lipids, Alabaster, AL, USA) loaded with 5 mM MES buffer (adjusted with Tris to pH 7.5), 0.25 mM K_2_SO_4_, 50.25 mM Na_2_SO_4_, 5 mM MgSO_4_. Liposomes were diluted into buffer at pH 7.5 using increasing K_2_SO_4_ concentrations, while lowering the concentration of Na_2_SO_4_ to retain the same osmolarity. Measurements were done in the presence of 50 nM oxonol VI and 100 nM valinomycin. The formation of a membrane potential was detected as the increase of dye fluorescence, which rapidly reached a maximum (F_max_) and then decreased due to what we attribute to be an inherent leakiness of the liposome preparation. Addition of 1 μM monensin collapsed the membrane potential and allowed the recording of a baseline fluorescence (F_0_) of the oxonol dye at 0 membrane potential. The value (F_max_ – F_0_)/F_0_ was plotted against the calculated membrane potentials and used for calibration.

The stoichiometry of KpNhaB WT was determined essentially as previously described for EcNhaB^18^. Briefly, for a strictly coupled antiporter that exchanges Na^+^ and H^+^, $\Delta {\tilde{\mu }}_{Na+}=\Delta {\tilde{\mu }}_{H+}$, or, using electrical units, ΔpNa + ΔΨ = n(ΔpH + ΔΨ). When ΔpH = 0, ΔpNa = (n − 1)ΔΨ.

Measurements using proteoliposomes were performed on KpNhaB WT proteoliposomes at a lipid to protein ratio of 100 loaded with different concentrations of Na_2_SO_4_ (using K_2_SO_4_ to compensate for osmolarity). KpNhaB D146A/D404A proteoliposomes were used at a lipid to protein ratio of 5. Nigericin (500 pM) was used throughout the proteoliposome measurements to dissipate the ΔpH formed through the activity of the exchanger^7^. To obtain a baseline of oxonol VI fluorescence, the protonophore SF6847 (Tyrphostin A9, Sigma Aldrich, Munich, Germany) was added at a concentration of 1 μM in the proteoliposome experiments in order to collapse the membrane potential. Under all conditions employed no membrane potential was generated when proteoliposomes were diluted in solution containing the same concentration of Na^+^ as the one inside the proteoliposomes, [Na^+^]_in_ = [Na^+^]_out_ (Supplementary Fig. S2).

## Supplementary information

Supplementary Information

## Data Availability

All data obtained in this study are included in this published article and the Supplementary Information.
