# Supplementary material for: Mutation of two key aspartate residues alters stoichiometry of the NhaB Na+/H+ exchanger from Klebsiella pneumoniae
Source: Sci Rep. 2019 Oct 28;9:15390. doi: 10.1038/s41598-019-51887-2 (PMC6817889; doi:10.1038/s41598-019-51887-2)
Supplement: Supplementary file 1 — Supplementary Information [file 41598_2019_51887_MOESM1_ESM.pdf]

Supplementary information

Mutation of two key aspartate residues alters stoichiometry of the NhaB Na^+/H^+ exchanger from *Klebsiella pneumoniae*

Miyer Patiño-Ruiz[1], Klaus Fendler[1], Octavian Călinescu[1,2*]

[1] Department of Biophysical Chemistry, Max Planck Institute of Biophysics, Frankfurt am Main, Germany

[2] Department of Biophysics, Carol Davila University of Medicine and Pharmacy, Bucharest, Romania

[*]Corresponding author, email: octavian.calinescu@umfcd.ro

Table S1. Sequence of the *Klebsiella pneumoniae* gene optimized for expression in *E. coli*.

ATGGAAATATCATATGGAAGGGCTTTATGGAGAAATTTTCTAGGGCAAAGTCCAGATTGGTACA
AACTGGCGCTGATTATTTTTCTGATTGTGAACCCGCTGGTGTTCGCGGTTGCGCCGTTTGTTGCGG
GTTGGCTGCTGGTTGTGGAGTTCATCTTTACCCTGGCGATGGCGCTGAAGTGCTACCCGCTGCTG
CCGGGTGGCCTGCTGGCGATCGAGGCGCTGCTGATTGGTATGACCAGCCCGGCGCACGTTCGTG
AGGAAATCGCGGGCAACCTGGAAGTGCTGCTGCTGCTGATCTTCATGGTTGCGGGTATTTATTTT
ATGAAGCAGCTGCTGCTGTTCGTGTTTACCCGTCTGCTGCTGGGCATTCGTAGCAAAATGCTGCT
GAGCCTGGCGTTCTGCCTGGCGGCGGCGTTCCTGAGCGCGTTTCTGGACGCGCTGACCGTGGTTG
CGGTGGTTATCAGCGTGGCGGTTGGTTTTTACGGCATTTATCACCGTGTTGCGAGCGCGCGTCCG
GACGATAACGATCTGCTGGACGATAGCCACATCGAACAGCACTACCGTGAGGTTCTGGAACAAT
TCCGTGGTTTTCTGCGTAGCCTGATGATGCATGCGGGTGTGGGTACCGCGCTGGGTGGCGTGATG
ACGATGGTGGGCGAGCCGCAGAACCTGATCATTGCGAAAGCGGCGGGTTGGCACTTCGGCGAGT
TCTTCATTCGTATGGCGCCGGTGACCGTTCCGGTGATGGTTTGCGGTCTGCTGACCTGCCTGCTG
GTTGAGAAGTACCGTCTGTTCGGTTATGGCGAACCGCTGCCGCCGACCGTGCGTAAAGTTCTGCA
GGACTTTGACGATCGTAGCCGTGCGCAACGTAGCCGTCAGGAGCAACTGCGTCTGCTGGCGCAA
GCGGTGATCGGCGTTTGGCTGATTGTTGCGCTGGCGTTCCACCTGGCGGAAGTGGGTCTGATCGG
CCTGAGCGTTATCATTCTGGCGACCACCTTTAGCGGTGTTACCGACGAGCACGCGATTGGCAAG
GCGTTCACCGAAGCGCTGCCGTTTACCGCGCTGCTGACCGTGTTCTTTGCGATCGTGGCGGTTAT
CATTGATCAGCAACTGTTCACCCCGGTGATTGAGTTTGTTCTGCAAGCGAGCCCGCACGCGCAAC
TGAGCCTGTTCTACCTGTTTAACGGTCTGCTGAGCAGCATCAGCGATAACGTGTTCGTTGGCACC
GTTTATATTAACGAGGCGAAAACCGCGCTGGAACACGGTGTGATCAGCCTGCCGCAGTTTGAAA
TGCTGGCGGTTGCGATTAACACCGGTACCAACCTGCCGAGCGTGGCGACCCCGAACGGTCAAGC
GGCGTTCCTGTTTCTGCTGACCAGCGCGCTGGCGCCGCTGATCCGTCTGAGCTACGGTCGTATGG
TGTGGATGGCGCTGCCGTATACCCTGGTGCTGACCCTGGTTGGCCTGCTGTGCGTGGAGTTCACC
CTGATGCCGGTTACCAACTGGCTGCTGGCGCACGGTTGGGTTACCACCCCGACCCTGCCGCACCA
TCACCATCACCACTAA

Table S2. Primers used for site-directed mutagenesis. Small letters denote the nucleotides differing from the template sequence.

Mutation	Primer_for	Primer_rev
D146A	CCTGAGCGCGTTTCTGGcCGCGCTG ACCGTGGTTGC	GCAACCACGGTCAGCGCGgCCAGA AACGCGCTCAGG
D146E	CCTGAGCGCGTTTCTGGAaGCGCTG ACCGTGGTTGC	GCAACCACGGTCAGCGCtTCCAGAA ACGCGCTCAGG
D404A	GGTCTGCTGAGCAGCATCAGCGcTA ACGTGTTCGTTGGCACCG	CGGTGCCAACGAACACGTTAgCGCT GATGCTGCTCAGCAGACC
D404E	GGTCTGCTGAGCAGCATCAGCGAg AACGTGTTCGTTGGCACCG	CGGTGCCAACGAACACGTTcTCGCT GATGCTGCTCAGCAGACC

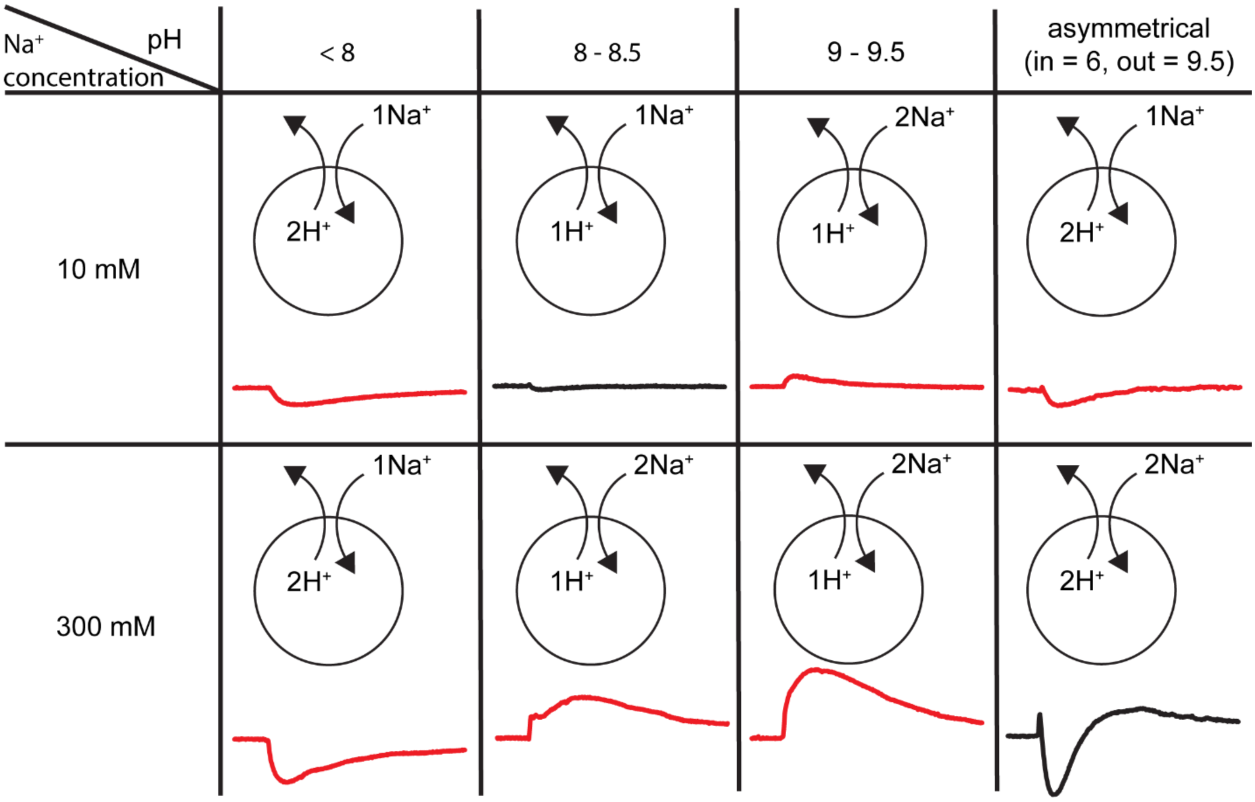

Figure S1. Transport modes of KpNhaB D146A/D404A observed using SSM-based electrophysiology. Arrows show the direction of substrate translocation. Traces in each panel are currents recorded under the respective conditions. Red traces denote conditions where steady-state electrogenic transport occurs.

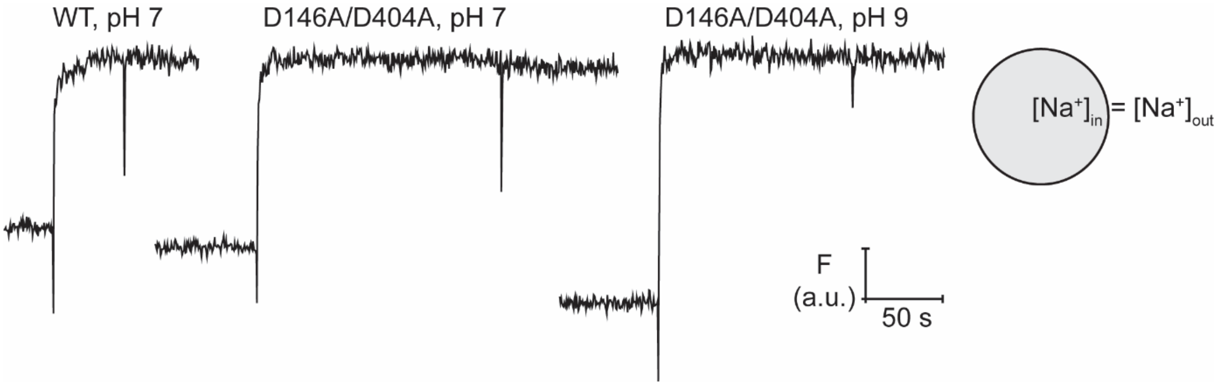

Figure S2. Fluorimetric measurements employing oxonol VI performed using KpNhaB proteoliposomes at symmetrical Na^+ concentration ($[Na^+]_{in} = [Na^+]_{out}$). Traces shown are representative of at least three independent measurements.
